# Auer “rhombuses”: The unusual cytomorphology of acute promyelocytic leukemia

**DOI:** 10.1002/jha2.628

**Published:** 2022-12-15

**Authors:** Vasily Ovechko, Matthew G. Bayes, Nekita Patel, Tejo Musunuru, Dingge Li, Kirill A. Lyapichev

**Affiliations:** ^1^ Department of Pathology The University of Texas Medical Branch Galveston Texas USA; ^2^ Department of Internal Medicine The University of Texas Medical Branch Galveston Texas USA

1

The patient is a 44‐year‐old inmate with a 3‐month history of unexplained pancytopenia. Prior medical history is significant for treated bipolar disorder and a substance use disorder involving synthetic cannabinoids (“K2”), along with paper soaked with pyrethroid containing pesticides (“KD”), since his arrival at a new facility 3 months ago. Laboratory studies showed a white cell count of 460/μl (reference range: 4200–10,700/μl), a hemoglobin level of 10.5 g/dl (reference range: 13.5–17.5 g/dl), and a platelet count of 28,000/μl (reference range: 150,000–400,000/μl). Peripheral blood smear examination was consistent with pancytopenia with no abnormal cells or blasts identified.

Bone marrow biopsy revealed trilineage hematopoiesis with rare promyelocytes containing numerous large rhomboid and smaller irregular shaped cytoplasmic immature pink azurophilic crystals (Wright–Giemsa stain, original magnification 1000×; Figure 1) forming clumps with cytoplasmic clearing. Although there were no traditional Auer rods identified, the suspicion for acute promyelocytic leukemia (APL) was raised following flow cytometric studies that showed a large aberrant myeloid population (65.3% of total events) of CD13^+^/CD33^+^/CD34^−^/CD64^+^/CD117^+^/CD123^+^/HLA‐DR^−^ cells consistent with an APL immunophenotype. A subsequent D‐dimer test was significantly elevated at 17.90 (<0.50 μg/ml (FEU)). Subsequent fluorescence in situ hybridization and karyotyping confirmed the presence of a t(15;17) PML‐RARA translocation. Treatment with arsenic trioxide (ATO) and all trans retinoic acid (ATRA) was initiated, and patient showed hematologic response with decreased D‐dimer values and improved cell counts.

**FIGURE 1:**
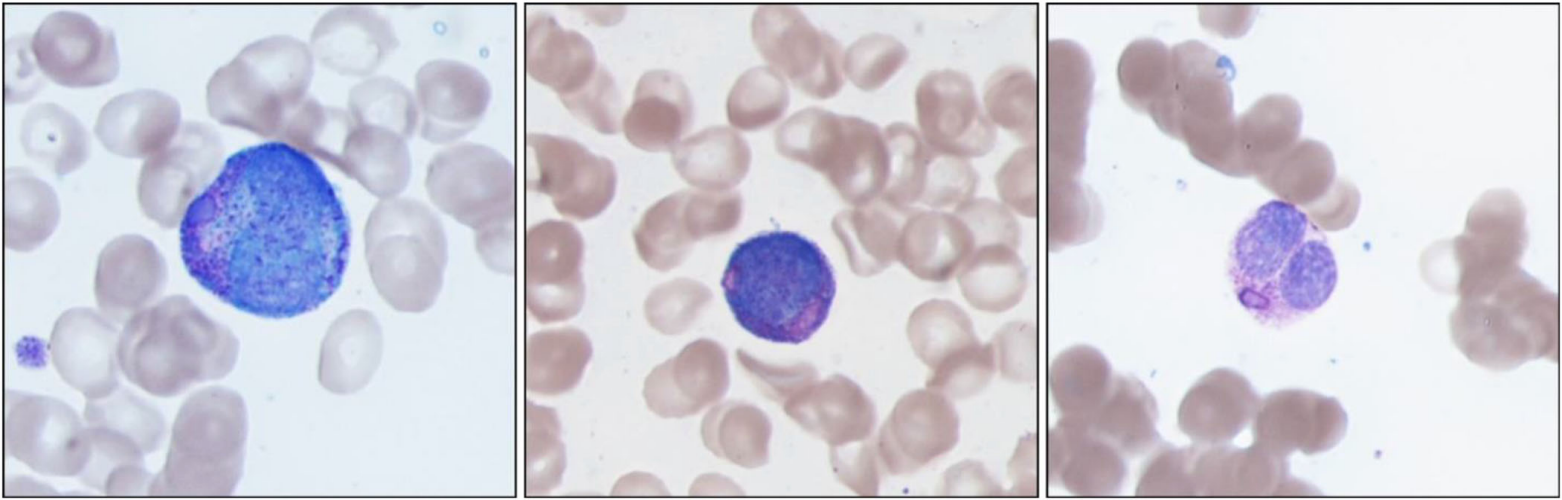
Promyelocytes containing numerous large rhomboid and smaller irregular shaped cytoplasmic immature pink azurophilic crystals (Wright–Giemsa stain, original magnification 1000×) forming clumps with cytoplasmic clearing

Review of literature shows similar, rare presentations of APL with Chediak–Higashi‐like granules instead of traditional Auer rods [1]. However, their occurrence in this patient with a unique presentation and history of substance abuse with novel synthetic cannabinoids and pyrethroid agents warrants attention as little is known about the prevalence of their use. One recent study showed that among people who use drugs, more than one in six people in rural Kentucky reported using pyrethroid agents via injection or smoking to get high over a period of 6 months [2] along with additional case reports of use and subsequent hospitalization [3].

## AUTHOR CONRIBUTIONS

Vasily Ovechko and Matthew G. Bayes wrote the draft (contributed equally); Nekita Patel and Tejo Musunuru provided invaluable feedback and major edits; Dingge Li provided invaluable feedback and flow cytometry analysis; Kirill A. Lyapichev provided the image, and conceived and designed the paper.

## CONFLICT OF INTEREST

The authors declare no conflict of interest.

## Data Availability

Data sharing not applicable to this image submission as no datasets were generated or analyzed.
